# Uptake of same-day initiation of HIV treatment in Malawi, South Africa, and Zambia as reported in routinely collected data: the SPRINT retrospective cohort study

**DOI:** 10.12688/gatesopenres.14424.2

**Published:** 2023-05-02

**Authors:** Amy Huber, Kamban Hirasen, Alana T. Brennan, Bevis Phiri, Timothy Tcherini, Lloyd Mulenga, Prudence Haimbe, Hilda Shakwelele, Rose Nyirenda, Bilaal Wilson Matola, Andrews Gunda, Sydney Rosen

**Affiliations:** 1 Health Economics and Epidemiology Research Office, Faculty of Health Sciences, University of the Witwatersrand, Johannesburg, Gauteng, 2193, South Africa; 2 Department Epidemiology, Boston University School of Public Health, Boston, MA, 02118, USA; 3 Department of Global Health, Boston University School of Public Health, Boston, MA, 02118, USA; 4 Clinton Health Access Institute-Zambia, Lusaka, Zambia; 5 Clinton Health Access Institute-Malawi, Lilongwe, Malawi; 6 Ministry of Health, Lusaka, Zambia; 7 Ministry of Health, Lilongwe, Malawi

**Keywords:** HIV, antiretroviral therapy, same-day initiation, Malawi, South Africa, Zambia

## Abstract

**Background: **Since 2017 global guidelines have recommended “same-day initiation” (SDI) of antiretroviral treatment (ART) for patients considered ready for treatment on the day of HIV diagnosis. Many countries have incorporated a SDI option into national guidelines, but SDI uptake is not well documented. We estimated average time to ART initiation at 12 public healthcare facilities in Malawi, five in South Africa, and 12 in Zambia.

**Methods: **We identified patients eligible to start ART between January 2018 and June 2019 from facility testing registers and reviewed their medical records from HIV diagnosis to the earlier date of treatment initiation or 6 months. We estimated the proportion of patients initiating ART on the same day or within 7, 14, 30, or 180 days of baseline.

**Results: **We enrolled 825 patients in Malawi, 534 in South Africa, and 1,984 in Zambia. Overall, 88% of patients in Malawi, 57% in South Africa, and 91% in Zambia received SDI. In Malawi, most who did not receive SDI had not initiated ART ≤6 months. In South Africa, an additional 13% initiated ≤1 week, but 21% had no record of initiation ≤6 months. Among those who did initiate within 6 months in Zambia, most started ≤1 week. There were no major differences by sex. WHO Stage III/IV and tuberculosis symptoms were associated with delays in ART initiation;  clinic size and having a CD4 count done were associated with an increased likelihood of SDI.

**Conclusions: **As of 2020, SDI of ART was widespread, if not nearly universal, in Malawi and Zambia but considerably less common in South Africa. Limitations of the study include pre-COVID-19 data that do not reflect pandemic adaptations and potentially missing data for Zambia. South Africa may be able to increase overall ART coverage by reducing numbers of patients who do not initiate ≤6 months.

## Introduction

In 2017, the World Health Organization (WHO) began recommending “same-day initiation” (SDI) of treatment for patients with HIV considered clinically and personally ready for antiretroviral therapy (ART) ^1^. This recommendation was based on a series of randomized trials and observational studies that had demonstrated improved retention in care and viral suppression rates for patients offered SDI, compared to those offered what was then the standard of care, which typically required multiple clinic visits before a patient received an initial supply of antiretroviral medications (ARVs) ^2^. Although determining a patient’s “readiness” for SDI ultimately relies on provider and patient discretion, reasons for delaying initiation include indications or suspicion of opportunistic infections such as tuberculosis or cryptococcal disease and a patient’s expressed desire for more time to accept the HIV diagnosis or disclose it to others ^2^.

In the clinical trials, most of the improvement in outcomes arose from a reduction in loss to follow up between testing positive for HIV and initiating ART. Observational studies have reported that among patients actually starting ART on the same day under routine care, loss to follow up during the first year after initiation has tended to be higher than in the pre-same day initiation period ^3, 4^, but these studies have generally compared outcomes among patients who started on the same day to outcomes among those known to have started within a specified number of days, ignoring patients who never started ART at all.

In response to the WHO recommendation, many countries adopted SDI into their national guidelines. Instructions to healthcare facilities as to exactly how to implement SDI and to which patients it should be offered, however, were not precise, allowing the interval between a patient’s first clinic visit and ART initiation to be determined by provider judgment and/or patient preference. Time intervals from a patient’s initial presentation (HIV diagnosis or first HIV-related healthcare interaction) to initiation of ART are not routinely reported in electronic medical record (EMR) systems, which typically only create a record for a patient starting at the time of ART initiation. Little evidence is available on the proportion of patients actually starting ART on the same day or the characteristics of those who do or do not, including age, sex, and other characteristics. Without this information, it is difficult to understand observed differences or identify opportunities for improving care.

This paper reports quantitative results of the SPRINT study (Survey of Procedures and Resources for Initiating Treatment of HIV in Africa). It documents uptake of SDI, time to ART initiation for those not receiving SDI, and predictors of SDI for adult men and women in three high HIV burden countries in sub-Saharan Africa.

## Methods

### Ethics

Ethical clearance was provided by the Boston University Medical Campus Institutional Review Board (IRB) (Ref No. H-40354 (Malawi, May 21, 2020), H-39330 (South Africa, October 15, 2019) and H-40488 (Zambia, July 10, 2020)), the University of the Witwatersrand Human Ethics Research Committee in South Africa (Ref No. M200238 (Malawi, July 10, 2020), M190745 (South Africa, November 6, 2019) and M200599 (Zambia, July 10, 2020)), the National Health Sciences Research Committee in Malawi (Ref No. 20/04/2458, April 24, 2020) and the ERES Converge Institutional Review Board in Zambia (Ref No. 2020-Feb-009, February 27, 2020). The Ministry or Department of Health in all three countries and the National Health Research Authority in Zambia also approved the study.

As this was a retrospective review of medical records and no identifiers were collected, a waiver of informed consent was provided. Data transcription from patient records began on May 27, 2020 in Malawi, September 28, 2020 in Zambia, and November 12, 2020 in South Africa.

IRB approval from the University of the Witwatersrand was sought to allow individuals on the team who were based in South Africa to access Malawi data for analysis purposes. There was no need for University of the Witwatersrand approval in order to collect the data, as that was only under the purview of the IRBs of Boston University and the National Health Sciences Research Committee in Malawi, whose approval was secured prior to the start of data collection. All required approvals were in place before data collection began in all three countries. Team members at the University of the Witwatersrand did not access the data from Malawi prior to their own institutional IRB approval.

### Study sites

SPRINT was a retrospective record review conducted in Malawi, South Africa, and Zambia. In each country, in collaboration with national health officials, we first purposively selected provinces and districts that provided diversity of setting (rural/urban) and of nongovernmental supporting partner organizations. We then identified a convenience sample of public sector primary healthcare clinics within these provinces or districts and conducted visits to these sites to assess facility-level and HIV testing and initiation-specific aggregate indicators. The final sites selected provided diversity in size and geographic setting, had relatively strong routine data collection, and were welcoming of the research team. Selected study sites are described in Table 1. All of the study sites offered universal treatment and followed national guidelines for ART treatment eligibility ^5– 7^; eligibility criteria included confirmed HIV-positive status and provider and patient concurrence that the patient was ready to start treatment, based on clinical and personal characteristics. The SPRINT studies are registered with Clinicaltrials.gov ( NCT04468399, July 13, 2020, Malawi; NCT04170374, Nov 20, 2019, South Africa; NCT04470011, July 14, 2020, Zambia). 

**Table 1.** SPRINT study sites.

Characteristics	Malawi	South Africa	Zambia
Number of sites	12	5 *	12
Geographic locations	Lilongwe District (Central Region), Blantyre District (Southern Region), Chiradzulu District (Southern Region)	Ehlanzeni District (Mpumalanga Province), King Cetshwayo District (KwaZulu-Natal Province)	Central Province, Lusaka Province
Settings			
Rural	7	3	4
Urban	5	2	8
Peri-urban	0	0	1
ART patient volume			
500–1,999	1	1	2
2,000–4,999	5	2	6
5,000–9,999	5	2	3
>10,000	1	0	1

*We originally collected enrollment data from eight sites in South Africa but were unable to access medical records from three of them, leaving a total of five study sites. SPRINT, Survey of Procedures and Resources for Initiating Treatment of HIV in Africa; ART, antiretroviral treatment.

At the time of study enrollment, guidelines for ART initiation in the three study countries encouraged but did not require that SDI be offered to patients whom providers determined to be eligible, without waiting for CD4 count results. In all countries, guidelines encouraged starting HIV treatment as soon as possible ^5– 7^ but also noted the potential need for delay among patients who indicated that they were not yet ready to start. The decision on whether a patient was eligible for same-day initiation was made by the authorized clinician, typically a clinical officer or trained ART nurse in Malawi and Zambia and a public health (senior) nurse in South Africa, each of whom was also authorized to prescribe antiretroviral medications once the decision to initiate was made.

Tuberculosis (TB) symptom screening was recommended in all three countries, with a TB test conducted for symptomatic patients prior to ART initiation. Malawi’s guidelines recommended TB symptom screening but encouraged ART initiation regardless of results: “[Start ART] within 14 days of diagnosis. TBT [TB treatment] + ART can be started on the same day if the patient is stable. Don’t delay TBT or ART.” Only South Africa’s guidelines explicitly required delaying ART initiation for all patients with TB symptoms, stating “Initiate ART after 2 weeks of TB treatment, when the client’s symptoms are improving, and TB treatment is tolerated.” Zambia’s guidelines recommended delaying ART based on suspicion of TB, without further elaboration. Guidelines did not specify actions for symptomatic patients who could not produce sputum samples for TB testing or for whom TB test results were not available, nor how long to delay ART initiation among patients diagnosed with active TB ^5– 7^.

### Enrollment and data collection

Patients were eligible for inclusion in the study if they were 1) non-pregnant adults (≥18 years old); 2) tested HIV-positive or provided documentation of HIV infection (prior positive test); and 3) were eligible to start or re-start ART at one of the study sites between 01 January to 31 December 2018 in South Africa or between 01 July 2018 and 30 June 2019 in Malawi and Zambia. We retrospectively identified up to 200 patients per site, starting on the latest date in the inclusion period (30 June 2019 in Malawi and Zambia, 31 December 2018 in South Africa) and working sequentially backward in time until the target sample size was reached (01 July 2018 in Malawi and Zambia, 01 January 2018 in South Africa). Study-eligible patients were selected from the HIV testing registers, testing database, or other testing records kept by the site, which were reviewed on site by study data collectors. Medical record data were downloaded from EMRs where available. Due to concerns about incomplete EMR data, however, all records for participants in Malawi and Zambia were extracted manually from paper files by study data collectors using a structured instrument and either the offline mobile or online web version of REDCap electronic data capture tools hosted at Wits University, depending on internet connectivity at the site. In South Africa, records were extracted manually at all facilities, but for 92 of 534 participants (17%) distributed across all the sites, paper files could not be found. For these records, EMR data were extracted instead.

Records were reviewed from the point of HIV treatment eligibility (HIV-positive diagnosis) to the point of treatment initiation or for a 6-month period, whichever was shorter. By starting with HIV diagnosis, rather than with treatment initiation, the study captured information about patients who failed to initiate treatment within 6 months (dropped out of care before being dispensed medications) and allowed us to observe services received and days elapsed before initiation. We note that the HIV test used to identify study participants may not have been any individual participant’s first positive test anywhere, but only their first positive test at the study site during the study period. Because repeat testing is common ^8^, we assume that many participants had previously tested positive at another location but failed to initiate treatment at that time or place.

### Sample size, outcomes, and statistical analysis

Study inclusion numbers were determined by resource availability. Our maximum sample size was 200 patients per site, or up to a total of 2,400 patients each in Malawi and Zambia and 1,600 in South Africa. The primary outcome of interest for the study was SDI, defined as being dispensed an initial supply of ARV medications on the day of testing positive for HIV or the day of first HIV-related clinic interaction, if a positive test had been conducted previously at another location, without subsequent linkage to care.

Simple descriptive statistics were used to describe the study sample. In order to assess site-specific characteristics, we stratified results by country, site, and presence of TB symptoms and ranked facilities by volume (number) of ART patients. Predictors of SDI were assessed using modified Poisson regression ^9^, clustered by study site to allow intragroup correlation in the standard errors. For our first model (Model 1) including data from all three countries, we adjusted for biological sex, age (categorized as 18–29, 30–39, 40–49 and ≥50 years of age), WHO stage III/IV ( *vs.* I/II), urban/peri-urban (vs. rural), volume on ART at each site, and country, all assessed at ART initiation, as potential risk factors for SDI. Considering that there were no CD4 cell count or TB symptom data from Malawi, we then ran the same model (Model 2) restricted to Zambia and South Africa and included a variable indicating whether the patient received a CD4 count (yes/no) and the presence of TB symptoms (yes/no) at initiation.

## Results

We identified a total 3,374 participants whose ART initiation dates were between 01 January to 31 December 2018 in South Africa or between 01 July 2018 and 30 June 2019 in Malawi and Zambia. After excluding 31 participants who were found to be ineligible (under 18 years old or initiated treatment outside the study period), the final analytic sample included 3,343 participants (825 patients in Malawi, 534 in South Africa, and 1984 in Zambia). We did not meet sample size targets due to resource limitations and COVID-19 restrictions that shortened the data collection period. CD4 counts and TB status were not available for patients in Malawi. There were no important differences in patient characteristics among the three countries, as shown in Table 2.

**Table 2.** Characteristics of the analytic study sample (n=3,343).

Characteristics (n (%))	Malawi		South Africa		Zambia	
Number enrolled	825	(100)	534	(100)	1,984	(100)
Age (years)						
18–29	269	(32.6)	172	(32.2)	656	(33.1)
30–39	308	(37.3)	217	(40.6)	781	(39.4)
40–49	164	(19.9)	100	(18.7)	389	(19.6)
50+	84	(10.2)	45	(8.4)	158	(8.0)
Sex (female)	428	(51.9)	301	(56.4)	1,015	(51.2)
WHO stage at ART initiation						
Stage III/IV	62 (7.5)		12 (2.3)		68 (3.4)	
Stage I/II	586 (71.0)		346 (64.8)		1,632 (82.3)	
WHO stage missing	177 (21.5)		176 (33.0)		284 (14.3)	
CD4 count at ART initiation						
Number with baseline CD4 count tests conducted	-		322 (60.3)		1,395 (70.3)	
Number with baseline CD4 count values recorded	-		283 (53.0)		470 (23.7)	
Median (IQR) (cells/mm ^3^)	-		302 (181-476)		378 (214-581)	
TB symptoms reported enrollment *						
Yes	-		62 (11.6)		327 (16.5)	
No	-		363 (68.0)		1,554 (78.3)	
Missing	-		109 (20.4)		103 (5.2)	

*The study was unable to collect data on reported TB symptoms in Malawi. WHO, World Health Organization; ART, antiretroviral treatment; TB, tuberculosis.

### Time to ART initiation

Time to ART initiation is presented in Table 3. Same-day initiation was the norm in Malawi (734/825, 89%) and Zambia (1,813/1,984, 91%), where a vast majority of patients did start ART on the day of diagnosis or first HIV-related clinic interaction. It was much less common in South Africa (304/534, 57%), where a fifth of patients (116, 22%) did not have a record of initiating ART at all within the 6-month study observation period. Among patients who did not initiate on the same day, varying proportions proceeded to initiate within 1, 2, 4, and 24 weeks, as shown in Table 3. Those who did initiate within 6 months, but not on the same day, made a median of 1 additional clinic visit (2 in total) between diagnosis and initiation (inclusive.).

**Table 3.** Time to ART initiation (n=3,344).

Time to ART initiation after HIV diagnosis (n (%))	Malawi			South Africa			Zambia		
	Male (n=397)	Female (n=428)	Total (n=825)	Male (n=233)	Female (n=301)	Total (n=534)	Male (n=969)	Female (n=1015)	Total * (n=1984)
Same-day initiation (0 days)	360 (90.7)	374 (87.4)	734 (89.0)	132 (56.7)	172 (57.1)	304 (56.9)	883 (91.1)	930 (91.6)	1813 (91.4)
Between 0 days and 6 months, of whom:	11 (2.8)	16 (3.7)	27 (3.3)	53 (22.7)	63 (20.9)	116 (21.7)	84 (8.7)	84 (8.3)	168 (8.5)
0 to ≤7 days	6 (1.5)	9 (2.1)	15 (1.8)	33 (14.2)	37 (12.3)	70 (13.1)	56 (5.8)	39 (3.8)	95 (4.8)
>7 to ≤14 days	2 (0.5)	2 (0.5)	4 (0.5)	3 (1.3)	9 (3.0)	12 (2.3)	12 (1.2)	22 (2.2)	34 (1.7)
>14 to ≤30 days	2 (0.5)	3 (0.7)	5 (0.6)	11 (4.7)	6 (2.0)	17 (3.2)	8 (0.8)	6 (0.6)	14 (0.7)
>30 days to ≤6 months	1 (0.3)	2 (0.5)	3 (0.4)	6 (2.6)	11 (3.7)	17 (3.2)	8 (0.8)	17 (1.7)	25 (1.3)
No initiation within 6 months	26 (6.6)	38 (8.9)	64 (7.8)	48 (20.6)	66 (21.9)	114 (21.4)	2 (0.2)	1 (0.1)	3 (0.2)
Median (IQR) number of clinic visits up to ART initiation for non-same day initiates (>day 0, ≤month 6 ^†^)	2 (2-3.5)	2 (2-2)	2 (2-3)	2 (2-2)	2 (2-2)	2 (2-2)	2 (2-2)	2 (2-2)	2 (2-2)

*As explained in text, the very small number of patients reported as not having initiated ART in Zambia may reflect missing data. ART, antiretroviral treatment. ^†^Includes visit at which ART was initiated.

Across all three countries, as shown in Table 3 and Table 4, we identified a total of 181 participants who did not initiate treatment within 6 months of HIV diagnosis or their first recorded HIV-related clinic visit (non-initiators). These patients comprised 8% (n=64) of the cohort in Malawi, 21% (n=114) in South Africa, and just 0.2% (n=3) in Zambia. We note that while these proportions are consistent with expectations in Malawi and South Africa, it is likely that non-initiators in Zambia who should have been included in our sample were not. Although we made multiple attempts to confirm the completeness of our cohort in Zambia, we believe it is likely that more than 0.2% of patients in Zambia who were eligible to initiate ART did not start within 6 months. Among those who did not initiate within 6 months, in Malawi most were either lost to follow-up or remained alive and in care, but not on ART, while in South Africa most were either lost to follow-up or were simply missing follow up records ( Table 4), a category which likely includes both unreported (silent) transfer to other healthcare facilities and loss to care.

**Table 4.** Outcomes for those who did not initiate (n=181).

6-month outcome for those who did not initiate within 6 months (n (%))	Malawi n=66	South Africa n=113	Zambia n=3	Total n=182
Alive and in care	20 (30.3)	4 (3.5)	-	24 (13.2)
Lost to follow up	1 (1.5)	1 (0.9)	-	2 (1.1)
Died	38 (57.6)	44 (38.9)	-	82 (45.1)
Transferred out	4 (6.1)	15 (13.3)	-	19 (10.4)
Missing (no follow up record)	3 (4.6)	49 (43.4)	3 (100)	55 (30.2)

When we stratified results by country, clinic, and TB symptom status and ranked sites according to ART patient volume (Supplemental Table 1), our results suggested that patients presenting with TB symptoms in South Africa were less likely to initiate ART on the same day than were those with TB symptoms. In rural clinics in South Africa, the likelihood of SDI tended to decline as patient volume increased. We did not see these associations in Zambia or Malawi.

### Predictors of same-day initiation

Table 5 presents the results of the modified Poisson regression. In Model 1, which included data from all three countries, we found that patients with a WHO stage III/IV ( *vs.* stage I/II) had an 8% decrease in the likelihood of SDI (adjusted risk ratio (aRR): 0.91; 95% confidence interval (CI): 0.85-0.98). Patients in South Africa had a 19% decrease in the likelihood of SDI (aRR: 0.81; 95% CI: 0.73-0.89) compared to Zambia, while the uptake of SDI in Malawi was comparable to that of Zambia. We did not control for CD4 counts or TB symptoms in this model, as these were not available in Malawi.

**Table 5.** Crude and adjusted models clustered by study site for the outcome of achieving SDI for the entire sample (Model 1) and for South Africa and Zambia where CD4 counts and TB data were collected (Model 2).

Characteristic	Measure	Model 1 SDI (n=3,343)		Model 2 SDI (n=2,518) *	
		Crude RR (95% CI)	Adjusted RR ^†^ (95% CI)	Crude RR (95% CI)	Adjusted RR† (95% CI)
Sex	Female	ref.	ref.	ref.	ref.
	Male	1.02 (0.98-1.05)	1.00 (0.98-1.02)	1.01 (0.97-1.05)	1.00 (0.97-1.02)
Age (years)	18-29.9	ref.	ref.	ref.	ref.
	30-39.9	1.00 (0.95-1.05)	0.98 (0.96-1.01)	0.98 (0.94-1.06)	0.98 (0.96-1.01)
	40-49.9	1.01 (0.96-1.07)	1.00 (0.97-1.03)	1.01 (0.94-1.09)	0.98 (0.95-1.01)
	≥50	1.00 (0.96-1.05)	1.01 (0.98-1.05)	1.02 (0.97-1.08)	1.01 (0.98-1.04)
WHO stage	I/II	ref.	ref.	ref.	ref.
	III/IV	0.94 (0.86-1.01)	0.91 (0.85-0.98)	0.86 (0.77-0.97)	0.92 (0.84-0.99)
Country	Zambia	ref.	ref.	ref.	ref.
	Malawi	0.97 (0.89-1.07)	1.08 (1.03-1.13)	-	-
	South Africa	0.62 (0.47-0.82)	0.81 (0.73-0.89)	0.62 (0.47-0.83)	0.82 (0.76-0.87)
Urban/peri-urban	No	ref.	ref.	ref.	ref.
	Yes	1.16 (0.97-1.38)	1.02 (0.96-1.09)	1.20 (0.90-1.61)	0.94 (0.88-1.00)
ART patient volume	500–2,000	ref.	ref.	ref.	ref.
	2,000–5,000	0.98 (0.81-1.18)	0.99 (0.92-1.07)	0.96 (0.76-1.21)	0.99 (0.93-1.04)
	5,000–10,000	1.01 (0.84-1.20)	1.05 (0.99-1.12)	1.04 (0.84-1.29)	1.09 (1.01-1.18)
	>10,000	1.05 (0.91-1.22)	0.99 (0.93-1.05)	1.07 (0.90-1.26)	1.07 (0.99-1.15)
TB symptoms	No	-	-	ref.	ref.
	Yes	-	-	0.79 (0.67-0.94)	0.85 (0.73-0.97)
CD4 count done	No	-	-	ref.	ref.
	Yes			1.07 (0.96-1.20)	1.10 (1.02-1.19)

*Restricted to South Africa and Zambia only ^†^Adjusted for biological sex, age (categorized as 18–29, 30–39, 40–49 and >50 years of age), WHO stage III/IV (vs. I/II) and country, all assessed at ART initiation.

In Model 2, restricted to South Africa and Zambia, we found similar results to Model 1. Patients with a WHO stage III/IV (vs. stage I/II) had an 8% decrease in the likelihood of SDI (aRR 0.92; 95% CI: 0.84-0.99) and those in South Africa had an 18% decrease in the likelihood of SDI compared to Zambia (aRR: 0.82; 95% CI: 0.76-0.87). We also found that those with TB symptoms had a 15% decrease in the likelihood of SDI (aRR 0.85; 95% CI: 0.73-0.97). Our results also suggest that patients who did have a CD4 count done (aRR: 1.10; 95% CI: 1.02-1.19) and larger vs. smaller clinic size (5,000-10,000 vs. <2,000 patients – aRR 1.09; 95% CI: 1.01-1.18 and >10,000 vs. <2,000 patients – aRR 1.07; 85% CI: 0.99-1.15) were more likely to initiate ART on the same day. Sex, age and location (urban/peri-urban or rural) were not predictors of SDI in either model. 

## Discussion

In this study, we found that by 2020, uptake of SDI was extensive in Zambia and Malawi, reaching 80–90% of patients initiating or re-initiating ART. It was much less common in South Africa, where just 57% of patients initiated on the same day. We did not find significant differences in time to initiation by sex; men were equally likely to start on the same day as women. Among patients who did not initiate same-day, roughly one third in Malawi, half in South Africa, and nearly all in Zambia went on to initiate within 6 months; the rest had no record of treatment initiation by the end of the 6-month follow-up period. Patients who initiated ART within 6 months but not on the day of diagnosis had a median of just two visits, including the initiation visit, suggesting that the initiation process has accelerated even outside SDI, with only one “pre-ART” visit occurring for most who do not receive SDI.

We found several other publications that reported the rate of SDI in various countries in sub-Saharan Africa, mostly at earlier time periods than our study, which was conducted between 2018 and 2020. A multi-country cohort study using data from The International epidemiology Databases to Evaluate AIDS (IeDEA) Network through 2018, including South Africa but not Malawi or Zambia, reported an overall rate of SDI of 64% ^10^. Three studies from South Africa were all completed roughly one year earlier than our study, in 2017–2018. The first, in Johannesburg, reported 20% uptake of SDI ^3^. The second, in Johannesburg and Limpopo Province, reported that 40% of patients received SDI, with an increase in uptake from 30.3% at the start of the study period to 54.2% at the end ^6^. The third, with data from four provinces, reported 54% SDI uptake in 2018, an increase from 18% in 2016 ^11^. The difference between these findings and our result of 57% SDI in South Africa in 2018–2020 likely reflects the impact of an additional year of facility experience in implementing the SDI guideline.

In Zambia, uptake of SDI increased from 42% to 75% of newly initiating patients between the beginning of 2016 and the beginning of 2018, a phenomenon attributed largely to the adoption of universal treatment eligibility in 2017 ^12^. By the time our data were collected in 2018–2020, Zambia’s SDI rate had increased to more than 91% ( Table 3). We did not find any published data on SDI uptake in Malawi to compare to our estimate of 88%. Reports from neighboring countries showed SDI rates of 65% in Zimbabwe ^7^ and 52% in Mozambique in 2017 ^13^; in Botswana uptake increased from 28% to 59% between 2018 and 2019 ^14^. SPRINT adds both geographic breadth and, importantly, more recent experience to these prior publications.

Reasons for uptake of SDI or of having delays in ART initiation in our study are not clear. In our data from South Africa and Zambia, only the presence of TB symptoms or a WHO stage of III or IV predicted a delay in initiation. This result likely reflects concerns about starting ART while patients may be experiencing co-infections, such as TB or cryptococcal meningitis. Global guidelines continue to regard these conditions as justification for delaying ART initiation ^15^, but recent studies suggest that many patients with TB symptoms can and should start ART immediately, as the risk of becoming lost to follow up if ART initiation is delayed may exceed the very low risk of experiencing TB immune reconstitution inflammatory syndrome ^16^. We note, however, that the timing of ART initiation in the presence of undiagnosed TB remains an area of active debate ^17^. Other reasons for delaying initiation may include clinicians’ individual views on SDI, patient preferences ( *e.g.*, concerns about stigma, disclosure, or side effects; feeling healthy or not in need of treatment ^18^), and/or lack of resolve or resources to adopt new SDI procedures at some facilities.

At the same time, valuable as SDI may be as a strategy for minimizing loss to follow up before starting ART treatment, it is likely that some patients should indeed delay initiation, as they require more urgent medical care before starting ART or have personal concerns about or barriers to treatment that cannot be addressed in a single visit ^19, 20^. The proportion of patients falling into this latter category is unclear, and it likely varies across different populations. Based on findings of the SLATE II trial in South Africa, a reasonable guess may be that 10–15% of patients are not good candidates for SDI ^16^. If this is correct, then both Zambia and Malawi have achieved near-universal uptake of SDI. South Africa still has some distance to go, however, before it has saturated demand for SDI.

The advent of COVID-19 as a major health risk in all three study countries as of the second quarter of 2020 is likely to have changed both patient and provider behavior with regard to ART initiation. On one hand, COVID-19 morbidity, fear of transmission, and societal restrictions kept many potential patients away from the clinic. In South Africa, for example HIV initiations are known to have fallen by 28% in the first year of the pandemic ^21^. Conversely, the desire to minimize personal interaction and clinic visits to avoid COVID-19 transmission could have favored SDI for patients who did make it to the clinic to start ART. It is also not clear whether provider and/or patient behavior will revert to earlier norms as pandemic restrictions wane or will sustain any changes that were made. A new round of data collection will be needed to answer those questions.

The SPRINT study had a number of limitations. Our final enrolled sample size was smaller than intended, due to resource limitations that shortened the data collection period. We relied entirely on routinely collected data, which resulted in many missing values for key variables. It is possible that the data sources we used for enrollment, such as HIV testing logs, missed some individuals who tested positive for HIV but did not register for HIV treatment. Despite every effort to enroll a representative sample of patients eligible for ART initiation, we are not confident that all patients who did not initiate ART during the study period were captured in Zambia, potentially leading to overestimates of total initiation in Zambia. Outcomes reported as “missing” in Table 4 may reflect unrecorded transfers to other healthcare facilities, rather than failure to initiate ART entirely, particularly in South Africa. Other country- and site-level differences in data completeness may also have contributed to the variations observed. Finally, we cannot discern from our data exactly why certain patients faced a delay in starting ART—whether it was a result of patient or provider preference, patient condition, social pressures, or some other barrier. We also cannot determine whether those who did not start on the same day were offered SDI and declined it or were simply not offered it at all. To achieve the full potential of SDI in increasing treatment uptake for those who are eligible and willing—and not creating risks for or coercing those who are not—a better understanding of how decisions are made at healthcare facilities would be of value.

## Data Availability

Data used in this study, which were abstracted from routinely collected medical records at public sector healthcare facilities, are owned by the Ministry of Health or Department of Health in each study country and cannot be shared by the authors. Requests for data access can be made to the national research authority of each study country. In Malawi, requests can be directed to the National Health Sciences Research Committee of the Ministry of Health and Population, P.O. Box 30377, Lilongwe 3, Malawi. In South Africa, requests can be directed to Mr. Xolile Ndzulu, National Health Information System ( ndzulx@health.gov.za, +27 12 395 8105). In Zambia, requests can be directed to the National Health Research Authority, P.O. Box 30075, Lusaka, Zambia. The authors may be able to advise on or offer updated data access information. Boston University Institutional Repository: Uptake of same-day initiation of HIV treatment among adult men and women in Malawi, South Africa, and Zambia: the SPRINT retrospective cohort study. https://hdl.handle.net/2144/46097 ^22^. This project contains the following extended data: Supplemental Table 1 (Proportion initiating same-day, stratified by country, clinic and TB symptom status and ranked by ART patient volume) Data are available under the terms of the Creative Commons Attribution 4.0 International license (CC-BY 4.0).
