# Supplementary material for: Uptake of same-day initiation of HIV treatment in Malawi, South Africa, and Zambia as reported in routinely collected data: the SPRINT retrospective cohort study
Source: Gates Open Res. 2023 May 2;7:42. Originally published 2023 Feb 14. [Version 2] doi: 10.12688/gatesopenres.14424.2 (PMC10160348; doi:10.12688/gatesopenres.14424.2)
Supplement: Peer review history

# Peer review: Reviewer response for version 2

**DOI:** 10.21956/gatesopenres.15981.r33131

**Published:** 2023-05-11

**Authors:** Rachael M. Burke

**Affiliations:** 1 London School of Hygiene & Tropical Medicine, London, UK; 2 Malawi Liverpool Wellcome, Blantyre, Malawi

Many thanks for your response. I have read the revised article and I have no further comments. These data are useful and important as we move to improving ART coverage.

Is the work clearly and accurately presented and does it cite the current literature?

Yes

If applicable, is the statistical analysis and its interpretation appropriate?

Partly

Are all the source data underlying the results available to ensure full reproducibility?

No source data required

Is the study design appropriate and is the work technically sound?

Yes

Are the conclusions drawn adequately supported by the results?

Yes

Are sufficient details of methods and analysis provided to allow replication by others?

Partly

Reviewer Expertise:

Infectious disease, epidemiology

I confirm that I have read this submission and believe that I have an appropriate level of expertise to confirm that it is of an acceptable scientific standard.

# Peer review: Reviewer response for version 2

**DOI:** 10.21956/gatesopenres.15981.r33133

**Published:** 2023-05-05

**Authors:** Richard Lessells

**Affiliations:** 1 KwaZulu-Natal Research Innovation & Sequencing Platform, University of KwaZulu-Natal, Durban, KwaZulu-Natal, South Africa

The authors have responded comprehensively to all queries/comments, and have made appropriate revisions to the manuscript. I have no follow-up queries.

Is the work clearly and accurately presented and does it cite the current literature?

Yes

If applicable, is the statistical analysis and its interpretation appropriate?

Yes

Are all the source data underlying the results available to ensure full reproducibility?

No

Is the study design appropriate and is the work technically sound?

Yes

Are the conclusions drawn adequately supported by the results?

Yes

Are sufficient details of methods and analysis provided to allow replication by others?

Partly

Reviewer Expertise:

Infectious diseases, HIV, clinical research

I confirm that I have read this submission and believe that I have an appropriate level of expertise to confirm that it is of an acceptable scientific standard.

# Peer review: Reviewer response for version 2

**DOI:** 10.21956/gatesopenres.15981.r33132

**Published:** 2023-05-04

**Authors:** Peter MacPherson

**Affiliations:** 1 University of Glasgow, Glasgow, UK

Thanks to the authors for a comprehensive set of responses. I have no further questions.

Is the work clearly and accurately presented and does it cite the current literature?

Yes

If applicable, is the statistical analysis and its interpretation appropriate?

Yes

Are all the source data underlying the results available to ensure full reproducibility?

No source data required

Is the study design appropriate and is the work technically sound?

Yes

Are the conclusions drawn adequately supported by the results?

Yes

Are sufficient details of methods and analysis provided to allow replication by others?

Yes

Reviewer Expertise:

Public Health, HIV, Tuberculosis, Epidemiology, Global Health

I confirm that I have read this submission and believe that I have an appropriate level of expertise to confirm that it is of an acceptable scientific standard.

# Peer review: Reviewer response for version 1

**DOI:** 10.21956/gatesopenres.15737.r32935

**Published:** 2023-04-04

**Authors:** Serena P. Koenig

**Affiliations:** 1 Brigham and Women’s Hospital, Harvard Medical School, Boston, MA, USA

Thank you for asking me to review this interesting and important paper, which presents routinely collected data on SDI in Malawi, South Africa, and Zambia. A total of 3344 records from 29 health facilities were recorded, which is lower than the target sample size of 6400 due to resource limitations. Overall, 91% of non-pregnant adults in Zambia, 88% in Malawi, and 57% in South Africa initiated SDI. Predictors of not initiating SDI were clinical Stage 3 or 4 disease, and TB symptoms.

 I have read the paper, and also the comments from 3 other reviewers. I agree with all comments by the other reviewers. This manuscript is clear and well written, and is an important contribution to the evidence base around SDI initiation. However, it’s difficult to know if differences in how data are captured and recorded could be contributing to the country-level differences which were found.

 I have included some additional details below, with the attempt at avoiding comments which have already been stated by the other reviewers:

Abstract:

 Methods:

1. I suggest substituting another phrase for “sequentially enrolled patients eligible to start ART” because this is a retrospective record review.
2. Please state the eligibility criteria to start ART.

Results:

1. Please describe the different study periods from South Africa, Malawi and Zambia. As it is currently written, it appears that this is 18 months in each country, but in the Methods section it clarifies that this is 12 months in each country, with different start and stopping periods. If possible, it would also be helpful to include the actual dates of inclusion, since it appears that these periods were shorter than 12 months (i.e. started at the last date in the 12-month period, and sequentially moved backward by day.
2. Please add numbers as well as percentages for those who were offered and accepted SDI. Do you also have the number of patients who were offered SDI and didn’t accept it?
3. For clarity, please give the numbers of patients who had not started SDI, but started within 7, 14, 30, or 180 days by country.
4. Please add p-values and confidence intervals for the predictors of SDI. 

Introduction: The last sentence states that this paper documents “update of SDI, time to ART initiation for those not accepting SDI, and predictors of SDI ….”  However, it appears that it is not possible to distinguish those who were not offered SDI from those who did not accept SDI. Please clarify this distinction.

Methods: Enrollment and data collection.

 I recommend changing the wording “patients were eligible for enrollment in the study if…” and “we sequentially enrolled up to 200 patients per site” as this is a retrospective chart review. 

Results:

1. I suggest changing the wording “we enrolled a total of 3374 participants” as this was a retrospective chart review, with a waiver of informed consent.
2. Time to ART Initiation: The first paragraph states that a third of patients in South Africa had not initiated ART within 6 months, but according to Table 3, this is 21.2%.  I would also suggest deleting this sentence, because in Table 4, it indicates that many of these non-initiators actually had missing follow-up data.
3. I agree with the review by Burke, in which she clearly the concerns about data quality, particularly for Zambia. I agree with comments and suggested approach in this regard. This is the most important critique of the paper.

Discussion: In Paragraph 4, I disagree with the statement “recent studies suggest that many patients with TB symptoms can and should start ART immediately, as the risk of becoming lost to follow up if ART initiation is delayed may exceed the very low risk of experiencing TB immune reconstitution inflammatory syndrome.” The authors should cite the systematic review by Burke *et al.* (2022 ^1^), which found insufficient evidence to recommend this approach. There are additional concerns about starting ART in the presence of undiagnosed TB, including post-TB lung disease.

Is the work clearly and accurately presented and does it cite the current literature?

Yes

If applicable, is the statistical analysis and its interpretation appropriate?

Partly

Are all the source data underlying the results available to ensure full reproducibility?

No source data required

Is the study design appropriate and is the work technically sound?

Yes

Are the conclusions drawn adequately supported by the results?

Yes

Are sufficient details of methods and analysis provided to allow replication by others?

Partly

Reviewer Expertise:

Infectious diseases, HIV, TB, clinical research

I confirm that I have read this submission and believe that I have an appropriate level of expertise to confirm that it is of an acceptable scientific standard, however I have significant reservations, as outlined above.

## References

[1] : Same-day antiretroviral therapy initiation for people living with HIV who have tuberculosis symptoms: a systematic review. *HIV Med* .2022;23(1) : 10.1111/hiv.13169 4-15 10.1111/hiv.13169 34528368

# Response

**Published:** 2023-04-14

**Authors:** Sydney Rosen

Thank you for asking me to review this interesting and important paper, which presents routinely collected data on SDI in Malawi, South Africa, and Zambia. A total of 3344 records from 29 health facilities were recorded, which is lower than the target sample size of 6400 due to resource limitations. Overall, 91% of non-pregnant adults in Zambia, 88% in Malawi, and 57% in South Africa initiated SDI. Predictors of not initiating SDI were clinical Stage 3 or 4 disease, and TB symptoms.

Response: We thank the reviewer for these comments.

- I have read the paper, and also the comments from 3 other reviewers. I agree with all comments by the other reviewers. This manuscript is clear and well written, and is an important contribution to the evidence base around SDI initiation. However, it’s difficult to know if differences in how data are captured and recorded could be contributing to the country-level differences which were found.

Response: We agree that it is hard to distinguish true differences from data discrepancies. Like virtually every other observational analysis of routine records in this region, our study almost certainly includes a mix of both. We believe, however, that it is still worth reporting the observed findings and discussing this problem in the manuscript. We have noted this in the limitations of the study.

 I have included some additional details below, with the attempt at avoiding comments which have already been stated by the other reviewers:

 Abstract:

 Methods:

- I suggest substituting another phrase for “sequentially enrolled patients eligible to start ART” because this is a retrospective record review.

Response: We have revised this phrase to “retrospectively identified.” This is the language suggested in https://journals.plos.org/plosmedicine/article/info:doi/10.1371/journal.pmed.0040297, the instructions for reporting cohort studies.

- Please state the eligibility criteria to start ART.

Response: Thank you for this suggestion. There is a limit of 300 words for the abstract, and we cannot add this information without exceeding that limit or replacing something that may be more important. We have therefore not included the eligibility criteria to start ART in the abstract but have confirmed that this information is included in the manuscript text with the description of the study sites.

 Results:

- Please describe the different study periods from South Africa, Malawi and Zambia. As it is currently written, it appears that this is 18 months in each country, but in the Methods section it clarifies that this is 12 months in each country, with different start and stopping periods. If possible, it would also be helpful to include the actual dates of inclusion, since it appears that these periods were shorter than 12 months (i.e. started at the last date in the 12-month period, and sequentially moved backward by day.

Response: We have added the actual dates of inclusion for each country. We assume that the reviewer refers to the period from January 2018 and June 2019 mentioned in the abstract as the 18 month study period. This is correct as the overall interval of ART initiation for participants in the study. The details for each country are provided in the text.

- Please add numbers as well as percentages for those who were offered and accepted SDI. Do you also have the number of patients who were offered SDI and didn’t accept it? 

Response: We have added numbers where there were previously only percentages. We have changed “offered and accepted” to “received,” as we do not know for certain that SDI was offered to those who did not start on the same day. It may have been offered but not accepted, or it may not have been offered.

- For clarity, please give the numbers of patients who had not started SDI, but started within 7, 14, 30, or 180 days by country.

Response: This information is reported in Table 3. We have avoided repeating it in the text to keep manuscript length under control.

- Please add p-values and confidence intervals for the predictors of SDI.

Response: Confidence intervals are reported in the text and in Table 5. P-values add no information for an observational study beyond that provided by confidence intervals and are widely misinterpreted. Based on American Statistical Association guidance ( https://amstat.tandfonline.com/doi/full/10.1080/00031305.2016.1154108#.Vt2XIOaE2MN%5D) we would therefore prefer not to report p-values for this work.

- Introduction: The last sentence states that this paper documents “update of SDI, time to ART initiation for those not accepting SDI, and predictors of SDI ….”  However, it appears that it is not possible to distinguish those who were not offered SDI from those who did not accept SDI. Please clarify this distinction.

Response: Thank you for this comment. We agree, we cannot make this distinction. We have thus changed the sentence from “accepting” to “receiving” SDI.

 Methods: Enrollment and data collection.

- I recommend changing the wording “patients were eligible for enrollment in the study if…” and “we sequentially enrolled up to 200 patients per site” as this is a retrospective chart review.

Response: We have revised this sentence.

 Results:

- I suggest changing the wording “we enrolled a total of 3374 participants” as this was a retrospective chart review, with a waiver of informed consent. 

Response: We have revised this sentence.

- Time to ART Initiation: The first paragraph states that a third of patients in South Africa had not initiated ART within 6 months, but according to Table 3, this is 21.2%.  I would also suggest deleting this sentence, because in Table 4, it indicates that many of these non-initiators actually had missing follow-up data.

Response: Thank you for catching the error in the first paragraph—it should have read “a fifth,” not a third. We have adjusted the language in the sentence to note that the result is based on records found.

- I agree with the review by Burke, in which she clearly the concerns about data quality, particularly for Zambia. I agree with comments and suggested approach in this regard. This is the most important critique of the paper.

Response: We agree about the importance of this issue and have addressed Dr. Burke’s comments above.

- Discussion: In Paragraph 4, I disagree with the statement “recent studies suggest that many patients with TB symptoms can and should start ART immediately, as the risk of becoming lost to follow up if ART initiation is delayed may exceed the very low risk of experiencing TB immune reconstitution inflammatory syndrome.” The authors should cite the systematic review by Burke et al. (20221), which found insufficient evidence to recommend this approach. There are additional concerns about starting ART in the presence of undiagnosed TB, including post-TB lung disease.

Response: We acknowledge that this is an ongoing debate in the literature and have added this point to the manuscript. Beyond that, we believe it is a judgment call, as some evidence supports and other evidence argues against this approach. Our statement “many patients can and should start ART immediately” appears to be correct for the setting in which our study took place, based on the reference cited. We did not say, and certainly do not believe, that all patients with TB symptoms should start ART immediately—only that many can and should, particularly in light of the high loss to follow up experienced between HIV testing and ART initiation at many of our sites. We would therefore like to keep this sentence in the manuscript. We have added the citation suggested, so that readers may pursue it if they wish.

# Peer review: Reviewer response for version 1

**DOI:** 10.21956/gatesopenres.15737.r32934

**Published:** 2023-03-17

**Authors:** Rachael M. Burke

**Affiliations:** 1 London School of Hygiene & Tropical Medicine, London, UK; 2 Malawi Liverpool Wellcome, Blantyre, Malawi

Many thanks for asking me to review. This paper presents information gathered from routine data sources on Same Day ART initiation in three Southern African countries. It is well reported and clear. These are important data for understanding programmatic ART implementation. I have read the paper and also the comments from the other two reviewers. I don't have much to add that the other reviewers haven't already mentioned.

1. To echo Dr Lessells, it would be helpful to know a bit more about why the sample size wasn’t reached in Malawi and Zambia? Did this introduce bias? Was this a smaller number of participants from all clinics? Or were some clinics under-represented? I see the discussion says results limitations, but it might be good if this were a little more detailed.
2. I agree with the other reviewers that it might be helpful to describe the data extraction process in a bit more detail by country and possibly by clinic. In what clinics were EMR data used vs. paper records? What happened if EMR data and paper data disagreed? (For example, same day ART recorded on one but not the other)? 
  What was the process of ascertaining HIV testing or first contact with systems? You say in the introduction often people are only entered into database at the point of starting ART (rather than point of first contact with system)? How were you able to get "one step" back from what is routinely reported?
  There is presumably an issue with under-ascertaining people who tested positive but didn’t ever start ART was a problem in Zambia (as the authors have said they don’t think their estimate of % who started ART is accurate), describing the data sources might help with understanding this.
  Was there a process of reconciling paper HIV testing registers with ART registers? How were similar-but-not-identical names or ages etc. dealt with? Do you think data collection processes at different clinics was similar?
  This information might be useful, particularly for understanding the Zambia context and how data on people who had a positive HIV test but didn’t initate might be lost.
  I do understand that this is all observational data from routine records, and some of these items might be very difficult to ascertain or not available at all. That is very understandable and doesn’t invalidate the research. Nonetheless, I think it would be good to describe a little further about the process of gathering/extracting data.
3. I agree with other reviewers that it might be helpful to see data broken down by clinic to see how much variation there is by clinic.
4. In table 5 from the model predicting SDI, is this comparing SDI vs. initiated ART (but not on the same day)? Or is it SDI vs. initiated not same day and not initiated at all? From the description of variables being collected at the point of ART initiation, it sounds like it is the former but it would be good to clarify.

In general, I agree with the comments from the other reviewers.

Minor comment

 I am not familiar with Zambia and South Africa ART guidelines, but the authors are correct in saying that in Malawi 2022 guidelines the timing of ART in people with diagnosed TB (or TB symptoms but not diagnosed TB) is not specified in detail.

Is the work clearly and accurately presented and does it cite the current literature?

Yes

If applicable, is the statistical analysis and its interpretation appropriate?

Partly

Are all the source data underlying the results available to ensure full reproducibility?

No source data required

Is the study design appropriate and is the work technically sound?

Yes

Are the conclusions drawn adequately supported by the results?

Yes

Are sufficient details of methods and analysis provided to allow replication by others?

Partly

Reviewer Expertise:

Infectious disease, epidemiology

I confirm that I have read this submission and believe that I have an appropriate level of expertise to confirm that it is of an acceptable scientific standard, however I have significant reservations, as outlined above.

# Response

**Published:** 2023-04-14

**Authors:** Sydney Rosen

Many thanks for asking me to review. This paper presents information gathered from routine data sources on Same Day ART initiation in three Southern African countries. It is well reported and clear. These are important data for understanding programmatic ART implementation. I have read the paper and also the comments from the other two reviewers. I don't have much to add that the other reviewers haven't already mentioned.

Response: We thank the reviewer for these comments.

- To echo Dr Lessells, it would be helpful to know a bit more about why the sample size wasn’t reached in Malawi and Zambia? Did this introduce bias? Was this a smaller number of participants from all clinics? Or were some clinics under-represented? I see the discussion says results limitations, but it might be good if this were a little more detailed.

Response: We have added mention of this at the start of the results section. We do not believe that this limitation introduced bias, as it resulted simply from slower data collection than expected (due in part to COVID-19 restrictions), which reduced the time available for the overall study. We enrolled sequentially from registers, which should produce a representative sample, even if smaller than we originally desired. (Supplemental Table 1, which we added in response to reviewers’ comments, indicates enrollment numbers for each study site.)

- I agree with the other reviewers that it might be helpful to describe the data extraction process in a bit more detail by country and possibly by clinic. In what clinics were EMR data used vs. paper records? What happened if EMR data and paper data disagreed? (For example, same day ART recorded on one but not the other)? 

Response: We have added further details about the data extraction process to the Methods section. We have clarified that we did not use both EMR and paper record data for any single patient, only one or the other. After reviewing the overall state of the EMR data for Malawi and Zambia, we decided to use manual data extraction from paper files only in these two countries. In South Africa, as explained above, we relied on EMR data only where paper files could not be found (lost or misfiled).

- What was the process of ascertaining HIV testing or first contact with systems? You say in the introduction often people are only entered into database at the point of starting ART (rather than point of first contact with system)? How were you able to get "one step" back from what is routinely reported?
  There is presumably an issue with under-ascertaining people who tested positive but didn’t ever start ART was a problem in Zambia (as the authors have said they don’t think their estimate of % who started ART is accurate), describing the data sources might help with understanding this.
  Was there a process of reconciling paper HIV testing registers with ART registers? How were similar-but-not-identical names or ages etc. dealt with? Do you think data collection processes at different clinics was similar?
  This information might be useful, particularly for understanding the Zambia context and how data on people who had a positive HIV test but didn’t imitate might be lost.
  I do understand that this is all observational data from routine records, and some of these items might be very difficult to ascertain or not available at all. That is very understandable and doesn’t invalidate the research. Nonetheless, I think it would be good to describe a little further about the process of gathering/extracting data.

Response: We thank the reviewer for understanding the challenges in using routine records. These are all really good questions. We don’t think it’s really possible to get “one step back” from routinely reported data in a retrospective, observational study. That said, as explained above, by starting with HIV testing, we went farther back than do most other studies of ART uptake. We believe that the HIV testing registers are relatively accurate and complete, though probably not perfect. In the end we did not enroll anyone who did not have a positive HIV test recorded in the HIV testing registers, which meant that all participants were enrolled as of the date of their HIV test. (We note that this might not have been a participant’s first positive HIV test ever, but it was the first at the study facility during the study period.)

 Matching HIV register patients to those in the ART registers was not a major concern, as the time period of interest for the ART registers was limited. Most patients initiated ART on the date of their HIV test, which allowed easy matching by date. For those who did not, we searched the ART registers and EMR records and matched by name and date of birth. We believe that for the sample enrolled this was likely an accurate reflection.

 The study had an established protocol, SOPs, and data collection forms that were used by all three countries and at all study sites. Where we had concerns about inconsistency, data collectors re-visited sites and re-checked registers and data. We do not know what happened in Zambia, or even if our speculation that some of those who tested positive and should have been enrolled were not captured. We just think it likely in view of the very high proportion of same-day initiation observed.

 In sum, we believe that the study has done everything possible to collect a complete data set for a representative sample of those eligible for same-day initiation. It is not perfect but we think it is better than has been done previously.

  

- I agree with other reviewers that it might be helpful to see data broken down by clinic to see how much variation there is by clinic.

Response: We have now reported results by facility in Supplemental Table 1. We h have also mentioned site-level variations in the Results section.

- In table 5 from the model predicting SDI, is this comparing SDI vs. initiated ART (but not on the same day)? Or is it SDI vs. initiated not same day and not initiated at all? From the description of variables being collected at the point of ART initiation, it sounds like it is the former but it would be good to clarify. In general, I agree with the comments from the other reviewers.

Response: The model compares SDI vs initiated not same day and not initiated at all (the latter option above). We created outcome variables of SDI = 1, anything else =0. This makes sense to us since the purpose of our analysis is identifying predictors of same-day initiation specifically, and the outcomes of those who did not initiate at all in our data set are unknown. As mentioned in the manuscript (now), some may have initiated at another facility, within 6 months but not same-day from our study site’s perspective.

Minor comment

- I am not familiar with Zambia and South Africa ART guidelines, but the authors are correct in saying that in Malawi 2022 guidelines the timing of ART in people with diagnosed TB (or TB symptoms but not diagnosed TB) is not specified in detail.

Response: Thank you, we also rechecked this in response to other reviewers’ questions.

# Peer review: Reviewer response for version 1

**DOI:** 10.21956/gatesopenres.15737.r32933

**Published:** 2023-03-07

**Authors:** Richard Lessells

**Affiliations:** 1 KwaZulu-Natal Research Innovation & Sequencing Platform, University of KwaZulu-Natal, Durban, KwaZulu-Natal, South Africa

This paper reports the main quantitative results from the SPRINT study, an observational study using routinely collected health data in Malawi, South Africa and Zambia. This study evaluated same-day antiretroviral therapy initiation for people with HIV in routine care settings. A total of 3344 records from 29 health facilities were analyzed (lower than target sample size of 6400 records due to resource limitations). Overall, the proportion of non-pregnant adults receiving same-day ART initiation was 91% in Zambia, 88% in Malawi and 57% in South Africa; and the proportion initiating ART within 6 months was 100% in Zambia, 92% in Malawi and 79% in South Africa. Individuals with clinical stage 3 or 4 disease and individuals with TB symptoms were less likely to receive same-day ART.

 This is an important contribution to the evidence base around same-day ART initiation, and adds to our understanding of how this is being implemented in HIV programmes in Africa. Despite not meeting the target sample size, the study still provides useful information that will be of interest to policy makers, programme managers and health care workers involved in HIV care delivery. Important differences between the three countries were found, with far fewer people receiving same-day ART at the South African sites. However, it’s difficult to know the extent to which this reflects true differences in practice, or whether differences in how data are captured/recorded could also contribute to these findings.  

 The manuscript is clear, concise and well-written. I have a few comments that the authors might want to consider, mainly highlighting areas where I think additional information or clarity would be helpful for the reader. 

Major comments:

1. As this is an observational study using routinely collected health data, I would recommend that reporting is in line with the RECORD statement and that the completed checklist is added as a supplementary file ( https://www.equator-network.org/reporting-guidelines/record/).
2. In the study registrations on clinicaltrials.gov, there are two primary outcome measures listed - Average number of visits required to start ART and Time to ART initiation. In this article, only Time to ART initiation has been reported. Please clarify if the protocol and primary outcomes were amended at some stage, or explain why the average number of visits required to start ART has not been reported.
3. For me, it’s critical to see more details of the data sources (e.g. electronic, register, files), and particularly what variables were used at each site/in each country to calculate the time to ART initiation. This seems key to understanding whether differences in data sources and data collection procedures might underlie any of the difference between sites/countries. Essentially it would be helpful to know how reliably the date of HIV testing or date of first engagement in care is captured, so that we know how confident we can be that if there is a true delay until ART initiation this will be reliably ascertained from the data source.

Minor comments:

1. Abstract, Methods: ‘We sequentially enrolled patients…’ sounds like this was a prospective cohort study where participants were enrolled. I would suggest rewording this to make clear that this was retrospective analysis of prospectively collected routine health data.
2. Abstract, Results: ‘…were offered and accepted SDI’ again is slightly misleading as it suggests you know more about who was offered SDI and who accepted this. Unless I’m mistaken, from the data available to you, you only know who received SDI.
3. Methods, Study sites: ‘Guidelines did not specify how long to delay ART initiation among patients diagnosed with active TB…’ This does not seem correct, as certainly SA guidelines stipulate when ART should be started for people being treated for active TB and I suspect this is also the case for guidelines in Zambia and Malawi. 
4. Methods, Study sites: It would be helpful to know for the three countries, or for the specific study sites, who was involved with ART prescribing/initiation. Was ART initiated by nurses at all sites, or did any sites require a doctor to initiate at the time of the study? This would give important context, especially to understanding any reason for lower proportion receiving SDI in South Africa.
5. Results: It would be good to see a table with the proportion receiving SDI by individual site/clinic – so that we can see how much variability there was, and particularly see whether the lower proportion was consistent across the five SA sites.
6. Results, Table 2: Were there no missing data for WHO stage?
7. Results, Table 2: Were there no missing data for TB symptoms at enrollment? Or does this just capture any records where there was positive documentation of TB symptoms? 
8. What was the reason that the actual CD4 count was not included in the model, and only whether or not the CD4 count was done/recorded? 
9. As the study specifically selected a mix of urban and rural sites, and higher/lower volume sites, was there a reason these were not included in the model – this might have helped to understand whether site characteristics were associated with SDI?
10. Discussion: The explanation about not meeting target sample size is only presented late in the discussion section. I think it would be good practice for this to be reported in the results.

Is the work clearly and accurately presented and does it cite the current literature?

Yes

If applicable, is the statistical analysis and its interpretation appropriate?

Yes

Are all the source data underlying the results available to ensure full reproducibility?

No

Is the study design appropriate and is the work technically sound?

Yes

Are the conclusions drawn adequately supported by the results?

Yes

Are sufficient details of methods and analysis provided to allow replication by others?

Partly

Reviewer Expertise:

Infectious diseases, HIV, clinical research

I confirm that I have read this submission and believe that I have an appropriate level of expertise to confirm that it is of an acceptable scientific standard, however I have significant reservations, as outlined above.

# Response

**Published:** 2023-04-14

**Authors:** Sydney Rosen

This paper reports the main quantitative results from the SPRINT study, an observational study using routinely collected health data in Malawi, South Africa and Zambia. This study evaluated same-day antiretroviral therapy initiation for people with HIV in routine care settings. A total of 3344 records from 29 health facilities were analyzed (lower than target sample size of 6400 records due to resource limitations). Overall, the proportion of non-pregnant adults receiving same-day ART initiation was 91% in Zambia, 88% in Malawi and 57% in South Africa; and the proportion initiating ART within 6 months was 100% in Zambia, 92% in Malawi and 79% in South Africa. Individuals with clinical stage 3 or 4 disease and individuals with TB symptoms were less likely to receive same-day ART.

 This is an important contribution to the evidence base around same-day ART initiation, and adds to our understanding of how this is being implemented in HIV programmes in Africa. Despite not meeting the target sample size, the study still provides useful information that will be of interest to policy makers, programme managers and health care workers involved in HIV care delivery. Important differences between the three countries were found, with far fewer people receiving same-day ART at the South African sites. However, it’s difficult to know the extent to which this reflects true differences in practice, or whether differences in how data are captured/recorded could also contribute to these findings.  

 The manuscript is clear, concise and well-written. I have a few comments that the authors might want to consider, mainly highlighting areas where I think additional information or clarity would be helpful for the reader. 

Response: We thank the reviewer for these comments.

Major comments:

- As this is an observational study using routinely collected health data, I would recommend that reporting is in line with the RECORD statement and that the completed checklist is added as a supplementary file ( https://www.equator-network.org/reporting-guidelines/record/).

Response: The RECORD checklist has been completed and will be posted to the Open BU repository ( www.open.bu.edu), as this journal does not accept supplementary files. (Editor: when the final page numbering is available, we will update the checklist and post it to the repository.)

- In the study registrations on clinicaltrials.gov, there are two primary outcome measures listed - Average number of visits required to start ART and Time to ART initiation. In this article, only Time to ART initiation has been reported. Please clarify if the protocol and primary outcomes were amended at some stage, or explain why the average number of visits required to start ART has not been reported.

Response: For this manuscript, we originally chose to report only one of the two primary outcomes in order to focus on the uptake of same-day initiation, with the intention to report visit numbers elsewhere. In response to the reviewer’s request, however, we now report visit numbers for those not initiating on the same day, as a final row in Table 3. The median for clients not initiating on the same day was two visits, including the visit at which ART was initiated.

- For me, it’s critical to see more details of the data sources (e.g. electronic, register, files), and particularly what variables were used at each site/in each country to calculate the time to ART initiation. This seems key to understanding whether differences in data sources and data collection procedures might underlie any of the difference between sites/countries. Essentially it would be helpful to know how reliably the date of HIV testing or date of first engagement in care is captured, so that we know how confident we can be that if there is a true delay until ART initiation this will be reliably ascertained from the data source.

Response: Dates of HIV testing were collected from clinic registers and records, as mentioned. We cannot say how reliable these data are, but in our experience, dates of discrete events like HIV tests are usually accurate. We did not mean to imply, however, that these dates necessarily reflect a participant’s first HIV test, or even first positive test, only the test associated with starting ART now. We have clarified this in the Methods section.

Minor comments:

- Abstract, Methods: ‘We sequentially enrolled patients…’ sounds like this was a prospective cohort study where participants were enrolled. I would suggest rewording this to make clear that this was retrospective analysis of prospectively collected routine health data.

Response: Thank you. We have reworded the abstract as suggested.

- Abstract, Results: ‘…were offered and accepted SDI’ again is slightly misleading as it suggests you know more about who was offered SDI and who accepted this. Unless I’m mistaken, from the data available to you, you only know who received SDI.

Response: Thank you. We have reworded the abstract as suggested.

- Methods, Study sites: ‘Guidelines did not specify how long to delay ART initiation among patients diagnosed with active TB…’ This does not seem correct, as certainly SA guidelines stipulate when ART should be started for people being treated for active TB and I suspect this is also the case for guidelines in Zambia and Malawi. 

Response: We agree that more detail in this regard may be important to the reader and have added it to the manuscript in the Methods section. In our view, however, none of the guidelines is definitive; even South Africa’s call for clinician judgment. South Africa’s guidelines at the time of the study stated “Initiate ART after 2 weeks of TB treatment, when the client’s symptoms are improving, and TB treatment is tolerated.” Zambia’s guidelines simply indicate that ART should be deferred for patients with TB. Malawi’s guidelines for patients with active TB state “Start ART within 14 days of diagnosis. TBT + ART can be started on the same day if the patient is stable. Don’t delay TBT or ART.”

 Please also see the response to Reviewer 1 with regard to delays due to TB.

- Methods, Study sites: It would be helpful to know for the three countries, or for the specific study sites, who was involved with ART prescribing/initiation. Was ART initiated by nurses at all sites, or did any sites require a doctor to initiate at the time of the study? This would give important context, especially to understanding any reason for lower proportion receiving SDI in South Africa.

Response: We have added this information to the Methods section. ART initiation, including prescribing, is conducted by clinical officers or trained ART nurses in Malawi and Zambia and by nurses in South Africa.

- Results: It would be good to see a table with the proportion receiving SDI by individual site/clinic – so that we can see how much variability there was, and particularly see whether the lower proportion was consistent across the five SA sites.

Response: We have added this information into Supplemental Table 1 and to the results section of the text.

- Results, Table 2: Were there no missing data for WHO stage? Were there no missing data for TB symptoms at enrollment? Or does this just capture any records where there was positive documentation of TB symptoms? 

Response: We have added the information on missingness for WHO stage and TB symptoms to Table 2.

- What was the reason that the actual CD4 count was not included in the model, and only whether or not the CD4 count was done/recorded? 

Response: Malawi did not routinely conduct CD4 count tests at initiation during the study period. In South Africa and Zambia (Model 2), data completeness for actual CD4 counts, rather than for whether a test was conducted or not, was poor. Actual CD4 count was recorded for only 751 of the 2,518 participants who had tests done. Rather than limiting Model 2 to only 751 subjects, we preferred to use the data for all 2,518. Additionally, when we controlled for CD4 count, we did not find it was a predictor of SDI. We have added a row to Table 2 to show the discrepancy between numbers of tests conducted and numbers of actual result values available.

- As the study specifically selected a mix of urban and rural sites, and higher/lower volume sites, was there a reason these were not included in the model – this might have helped to understand whether site characteristics were associated with SDI?

Response: We have added setting (urban/peri-urban and rural) and total remaining on ART (TROA) categorized into 4 groups (500-2,000, 2,000-5,000, 5,000-10,000 and >10,000) to both models (Table 5) and to Table 1. Only 1 site was peri-urban in Zambia, so we grouped urban/peri-urban settings together. We have added the following text to the results section at the bottom of page 13 in reference to model 2 results, “…larger vs. smaller clinic size (5,000-10,000 vs. <2,000 patients – aRR 1.09; 95% CI: 1.01-1.18 and >10,000 vs. <2,000 patients – aRR 1.07; 85% CI: 0.99-1.15) were more likely to initiate ART on the same day. Sex, age and location (urban, peri-urban or rural) were not predictors of SDI in either model.”

- Discussion: The explanation about not meeting target sample size is only presented late in the discussion section. I think it would be good practice for this to be reported in the results.

Response: We have now noted this in the first paragraph of the Results section.

# Peer review: Reviewer response for version 1

**DOI:** 10.21956/gatesopenres.15737.r32932

**Published:** 2023-02-22

**Authors:** Peter MacPherson

**Affiliations:** 1 University of Glasgow, Glasgow, UK

Many thanks for asking me to review this manuscript. Great to see these data, and very clearly reported. I have only a few comments that the authors should address.

1. In the introduction, it might be worth adding a sentence or two explaining some of the reasons why same day ART initiation may not always be possible or recommended.
2. Page 3, in the paragraph describing country guidelines for SDI. Did any country guidelines have recommendations for ART initiation in people with TB symptoms who were not able to produce sputum samples, or did not receive results? This is likely a common reason for ART initiation delay.
3. Methods: good to provide assurance that, in each of the study clinics, people were systematically recorded in registers/records at the point of eligibility assessment. i.e. is it possible that some people who attended the clinic, but felt to be not eligible for ART initiation by staff, did not have their attendance recorded? If this was a possibility, did practice vary by clinic/country, and what biasing effect may it have had on estimates of same-day initiation?
4. Similarly, in ascertaining outcomes (ART initiation as defined by dispensing of an initial supply of medication), did recording practices differ between sites/countries, and what methods were put in place to ensure ascertainment bias was minimised?
5. In Table 1, would it be possible to add a row to provide the number of clinics per country from which electronic data records were available for extraction?
6. Table 2: interesting to see that the prevalence of reported TB symptoms was lower in the South African clinics compared to the Zambian clinics. Were there any differences in definitions of “TB symptoms” or recording between countries?
7. Table 4 - do you have data on the timing of death?
8. Great to see that clinic-level random intercepts were included in the modified Poisson regression models, if I am understanding correctly. Are you able to quantify the variation in percentage achieving same-day initiation between clinics (e.g. standard deviation of the random intercepts), and any particular patterns that might help explain why clinics had higher or lower rates of same-day initiation (e.g. urban vs. rural clinics, size of clinic)? Investigating this may help untangle some of the potential reasons for delay rightly highlighted by the authors as a remaining knowledge gap in the Discussion.

Is the work clearly and accurately presented and does it cite the current literature?

Yes

If applicable, is the statistical analysis and its interpretation appropriate?

Yes

Are all the source data underlying the results available to ensure full reproducibility?

No source data required

Is the study design appropriate and is the work technically sound?

Yes

Are the conclusions drawn adequately supported by the results?

Yes

Are sufficient details of methods and analysis provided to allow replication by others?

Yes

Reviewer Expertise:

Public Health, HIV, Tuberculosis, Epidemiology, Global Health

I confirm that I have read this submission and believe that I have an appropriate level of expertise to confirm that it is of an acceptable scientific standard, however I have significant reservations, as outlined above.

# Response

**Published:** 2023-04-13

**Authors:** Sydney Rosen

Many thanks for asking me to review this manuscript. Great to see these data, and very clearly reported. I have only a few comments that the authors should address.

Response: We thank the reviewer for these comments.

  

- In the introduction, it might be worth adding a sentence or two explaining some of the reasons why same day ART initiation may not always be possible or recommended.

Response: Thank you for this suggestion. We have added a sentence about this to the introduction.

  

- Page 3, in the paragraph describing country guidelines for SDI. Did any country guidelines have recommendations for ART initiation in people with TB symptoms who were not able to produce sputum samples, or did not receive results? This is likely a common reason for ART initiation delay.

Response: This is a good point. We agree that concerns about undiagnosed TB have been a major reason for delaying ART initiation. South Africa’s guidelines explicitly exclude patients with a positive TB symptom screen from the recommendation for same-day initiation. Malawi’s guidelines do require TB symptom screening but do not explicitly recommend delaying ART initiation on the basis for the screening results. Zambia’s guidelines recommend deferring initiation if there is suspicion of TB but do not specify what should be considered for “suspicion.” We have added this information to the Methods section where the guidelines are described.

- Methods: good to provide assurance that, in each of the study clinics, people were systematically recorded in registers/records at the point of eligibility assessment. i.e. is it possible that some people who attended the clinic, but felt to be not eligible for ART initiation by staff, did not have their attendance recorded? If this was a possibility, did practice vary by clinic/country, and what biasing effect may it have had on estimates of same-day initiation

Response: Another good point. It is true that recording of patients not eligible for ART initiation is poor in many clinics in the study countries. This is the reason for enrolling SPRINT participants at the point of testing positive for HIV, which is the very first step in the ART eligibility determination process. Even patients who arrive with documentation of a positive HIV test from somewhere else are re-tested so that the facility has confirmation of their status. SPRINT enrolled on the basis of HIV testing registers and then followed patients forward in time. We did not enroll anyone who was not tested at the study clinic, though there was no requirement that the test used for study enrollment was the individual’s first positive HIV test—a prior test at any time in the past was certainly possible. While we cannot be certain that every individual who tested positive was included in the registers, we believe that the study captured as many of them as is possible in a retrospective analysis. We have clarified the wording about this in the methods section and, since missing some positive testers is a possibility, we added a note about this to the study limitations section.

- Similarly, in ascertaining outcomes (ART initiation as defined by dispensing of an initial supply of medication), did recording practices differ between sites/countries, and what methods were put in place to ensure ascertainment bias was minimized?

Response: Ascertainment bias is always a possibility when relying on routinely collected data, as the reviewer suggests. Prescribing medications is usually one of the best-recorded indicators in routinely kept medical records, however, so we are not too concerned about bias in this regard. A more serious concern arising from our reliance on routinely collected records within unlinked EMRs is that patients who appeared not to have initiated ART at all during the six-month follow up period (last row of Table 4) could actually have transferred informally (“silent transfers”) to other healthcare facilities. The number of such participants is very low in Zambia and Malawi but this may explain the larger number of missing outcomes in South Africa, where mobility is high. We have added this explanation to the results section and the limitations section in the discussion.

-  In Table 1, would it be possible to add a row to provide the number of clinics per country from which electronic data records were available for extraction?

Response: Since Table 1 describes the study sites, we assume that this question pertains to the study sites and not the study countries as a whole. All study sites had electronic data records available for extraction, but none of the study sites reliably entered into their EMRs patients who did not start ART. We therefore relied entirely on paper record data extraction in Zambia and Malawi, where the EMRs were of greater concern. In South Africa, we used EMR data to supplement paper records for all 5 clinics, as we were not able to find all paper files (EMR used for 92 of 534 participants, 17%). In these cases, the paper records were likely misfiled or misplaced after original EMR data entry by the clinic. We have added these details to the manuscript.

- Table 2: interesting to see that the prevalence of reported TB symptoms was lower in the South African clinics compared to the Zambian clinics. Were there any differences in definitions of “TB symptoms” or recording between countries?

Response: We agree that this was a bit surprising, given what we think we know about the prevalence of TB in the two countries, but we have no way of answering the reviewer’s questions. Both countries use the standard WHO four-symptom screening tool. It is possible that Zambian clinic staff are more diligent about recording results, but we have no evidence of this. Since we don’t know, and cannot even really guess, we’d prefer not to speculate in the manuscript.

- Table 4 - do you have data on the timing of death?

Response: Unfortunately, date of death is rarely recorded in routine ART clinic records, so we do not.

- Great to see that clinic-level random intercepts were included in the modified Poisson regression models, if I am understanding correctly. Are you able to quantify the variation in percentage achieving same-day initiation between clinics (e.g. standard deviation of the random intercepts), and any particular patterns that might help explain why clinics had higher or lower rates of same-day initiation (e.g. urban vs. rural clinics, size of clinic)? Investigating this may help untangle some of the potential reasons for delay rightly highlighted by the authors as a remaining knowledge gap in the Discussion.

Response: We thank the reviewer for this suggestion. We only clustered by clinic, so we are unable to quantify the variation in percentage achieving SDI between clinics. We felt it would be informative to stratify uptake of SDI by country, site location (urban/peri-urban and rural) and tuberculosis symptoms present and to rank them by size of clinic based on the total volume of patients on ART at each site.

 We have added Supplemental Table 1 below with the facility-level results for these variables to the manuscript. The following text into the results section in reference to any variation by site, “When we stratified results by country, clinic, and TB symptom status and ranked sites according to ART patient volume (Supplemental Table 1), our results suggested that patients presenting with TB symptoms in South Africa were less likely to initiate ART on the same day than were those with TB symptoms. In rural clinics in South Africa, the likelihood of SDI tended to decline as patient volume increased. We did not see these associations in Zambia or Malawi.”

 (Please note that we cannot include tables in these responses. Supplemental Table 1 will be submitted to the journal with the revised manuscript.)

 Additionally, we have added setting (urban/peri-urban and rural) and volume on ART, categorized into 4 groups (500-2,000, 2,000-5,000, 5,000-10,000 and >10,000), to both models (Table 5) and to Table 1. Only 1 site was peri-urban in Zambia, so we grouped urban/peri-urban settings together. We have added the following text to the results section at the bottom of page 13 in reference to model 2 results, “…larger vs. smaller clinic size (5,000-10,000 vs. <2,000 patients – aRR 1.09; 95% CI: 1.01-1.18 and >10,000 vs. <2,000 patients – aRR 1.07; 85% CI: 0.99-1.15) were more likely to initiate ART on the same day. Sex, age and location (urban, peri-urban or rural) were not predictors of SDI in either model.”
